# Determinants of myocardial fibrosis in patients with immune-mediated inflammatory diseases

**DOI:** 10.1186/s42358-025-00451-w

**Published:** 2025-06-14

**Authors:** Nicholas Black, Joshua Bradley, Fardad Soltani, John P. Farrant, Josephine H. Naish, Matthias Schmitt, Maya H. Buch, Christopher A. Miller

**Affiliations:** 1 Division of Cardiovascular Sciences, School of Medical Sciences, Faculty of Biology, Medicine and Health, Manchester Academic Health Science Centre, University of Manchester, Oxford Road, Manchester, M13 9PL UK; 2 Manchester University NHS Foundation Trust, Southmoor Road, Wythenshawe, Manchester, M23 9LT UK; 3 Division of Musculoskeletal and Dermatological Sciences, School of Medical Sciences, Faculty of Biology, Medicine and Health, Manchester Academic Health Science Centre, University of Manchester, Oxford Road, Manchester, M13 9PL UK; 4 NIHR Manchester Biomedical Research Centre, Manchester Academic Health Science Centre, Manchester University NHS Foundation Trust, University of Manchester, Manchester, UK

**Keywords:** Myocardial fibrosis, Extracellular volume, Immune-mediated inflammatory disease

## Abstract

**Background:**

Myocardial fibrosis is an important adverse prognostic marker, however; determinants of myocardial fibrosis in immune-mediated inflammatory diseases (IMIDs) remain poorly defined. We aimed to identify determinants of myocardial fibrosis in patients with IMIDs, as measured by cardiovascular magnetic resonance (CMR) extracellular volume (ECV).

**Methods:**

Cross-sectional study of 116 patients with IMIDs undergoing clinical CMR at Manchester University NHS Foundation Trust. IMIDs included rheumatoid arthritis, systemic lupus erythematosus, systemic sclerosis (SSc), ankylosing spondylitis, psoriatic arthritis and vasculitis. CMR included pre- and post-contrast T1 mapping to measure myocardial ECV, with same day blood sampling. Determinants of ECV were investigated with univariable and multivariable linear regression.

**Results:**

ECV varied significantly according to IMID diagnosis (ANOVA F statistic 2.80, *P* = 0.015); ECV was higher in patients with SSc compared to other IMIDs. Major determinants of ECV as a continuous variable were SSc, smoking and body mass index (BMI); regression coefficients 3.33 (95% confidence interval 0.82–5.84), 3.08 (0.73–5.43), and − 0.19 (-0.29 – -0.09) respectively, *P* < 0.01 (SSc, smoking and lower BMI were associated with increased ECV). Approximately a quarter of the variability in ECV could be explained by these predictors (optimism adjusted R^2^ 0.265).

**Conclusion:**

SSc is associated with a higher burden of myocardial fibrosis compared to other IMIDs. In patients with IMIDs, independent determinants of myocardial fibrosis were presence of SSc, smoking and BMI. Importantly, participants underwent CMR for clinical indications and may not be representative of IMID populations in the community.

**Supplementary Information:**

The online version contains supplementary material available at 10.1186/s42358-025-00451-w.

## Background

Immune-mediated inflammatory diseases (IMIDs) are a diverse group of diseases with overlapping clinical features and immunopathogenesis [1]. Examples include rheumatoid arthritis, systemic lupus erythematosus (SLE), systemic sclerosis (SSc), ankylosing spondylitis, psoriatic arthritis and vasculitis. Over 1 million people in the UK are diagnosed with an IMID [2]. Cardiovascular disease remains the most common cause of excess death in patients with IMIDs [3]. IMIDs may cause cardiac disease through direct organ involvement i.e., myocarditis and pericarditis, and are associated with accelerated coronary artery disease [3, 4].

Cardiovascular magnetic resonance (CMR) imaging has provided new insights into cardiovascular disease mechanisms. Late gadolinium enhancement (LGE) imaging has identified a high prevalence of non-ischaemic focal myocardial fibrosis, and unrecognised myocardial infarctions in IMID populations [5–10].

CMR extracellular volume (ECV) provides accurate and robust measurement of myocardial fibrosis [11], and is a powerful determinant of adverse outcomes, such as heart failure and death, in a wide range of conditions, including non-ischaemic cardiomyopathies, ischaemic heart disease, valvular heart disease and heart failure [12–14]. Although previous studies have shown ECV to be significantly increased in SSc [7, 15], the relationship between ECV and other IMIDs is less well characterised. Moreover, mechanisms underlying myocardial fibrosis in IMIDs remains poorly understood. In the current study, we sought to compare ECV between IMIDs and identify determinants of ECV in the IMID population.

## Methods

### Study population

Between 5th January 2015 and 17th May 2021, consecutive patients undergoing clinical CMR at Manchester University NHS Foundation Trust, United Kingdom were prospectively recruited (NCT02326324). Patients were assessed for the presence of IMIDs through questionnaire screening and medical record confirmation, and those with confirmed rheumatoid arthritis, SLE, SSc, ankylosing spondylitis, psoriatic arthritis, or vasculitis (classified as large, medium or small-vessel vasculitis) were included in the current analysis. The investigation conforms with the *Declaration of Helsinki.* The study was approved by a NHS Research Ethics Committee, and all participants provided written informed consent.

### Procedures

Data were managed using Research Electronic Data Capture (REDCap) hosted at Manchester University NHS Foundation Trust [16]. Baseline clinical data were determined from study questionnaire and medical records. CMR imaging was performed using two scanners (1·5T Avanto, and 3T Skyra; Siemens Medical Imaging). Scanning included steady-state free precession cine imaging in standard long- and short-axis planes, basal and mid LV short-axis T1 mapping (MOdified Look-Locker Inversion Recovery) before and after gadolinium-based contrast agent (gadoterate meglumine [Dotarem; Guerbet, France]), and LGE imaging. Image analysis was performed using cvi42 (version 5.6.7; Circle Cardiovascular Imaging; Calgary, Canada) in line with Societal Recommendations as described previously [17, 18]. Global longitudinal strain was measured as described previously [19]. High sensitivity troponin T (hsTnT), N-terminal pro-B-type natriuretic peptide (NT-proBNP) and growth differentiation factor-15 (GDF-15) (cobas e411 immunoanalyser, Roche Diagnostic, UK), as well as haematocrit, were laboratory assessed from blood sampling performed on the same day as CMR.

### Statistical analysis

All analyses were performed using R (Version 4.2.0, R Foundation for Statistical Computing, Austria). The overall percentage of missing data was 11.4% with 75% of patients having at least one missing data point (Supplementary Table 1). Missing data were handled using multiple imputation with chained equations [20]. 75 imputed datasets were generated with satisfactory imputation performance (Supplementary Figs. 1–3). Estimates were combined and pooled according to current guidelines [21, 22]. Optimism adjusted R^2^ was calculated by bootstrapping the imputed datasets 200 times and refitting the pooled model. Natural logarithmic *(ln)* transformation of hsTnT, NT-proBNP and GDF-15 were performed because of non-normal distributions. CMR data from two patients were excluded as outliers (severely dilated hearts due to congenital heart disease and known dilated cardiomyopathy, respectively). One-way analysis of variance (ANOVA) was used to assess for a significant difference in mean ECV between IMID diagnoses by calculating the F-statistic for the ratio of between to within sample variance. A post hoc Tukey test was used for multiple pairwise comparison of mean ECV between IMID diagnoses. A receiver operating characteristic (ROC) curve was constructed for the sensitivity and specificity of ECV for classifying SSc vs. other IMIDs. The optimal ECV threshold was determined using the Youden Index. Univariable and multivariable linear regression (backward stepwise selection) were used to determine the relationships between baseline variables and ECV [20]. Model assumptions were assessed on imputed datasets (Supplementary Fig. 4). Unless otherwise stated, values are given as mean ± standard deviation.

## Results

### Patient characteristics

The cohort comprised 116 patients with a confirmed IMID diagnosis, including 57 patients with rheumatoid arthritis, 15 with SLE, 11 with SSc (4 limited form), 11 with ankylosing spondylitis, 11 with psoriatic arthritis and 11 with vasculitis (1 large vessel (Takayasu arteritis), 1 medium vessel (polyarteritis nodosa), 7 small vessel (6 ANCA-associated vasculitis, 1 leukocytoclastic vasculitis), and 2 cases of Behcet’s disease). Baseline characteristics are summarised in Table 1. Mean age was 59 ± 14 years and 72 (62%) were female. Indications for CMR scan are summarised in Supplementary Table 2.

**Table 1:** Baseline characteristics

	Overall (*N* = 116)	Rheumatoid arthritis (*N* = 57)	Systemic lupus erythematosus (*N* = 15)	Systemic sclerosis^Δ^ (*N* = 11)	Ankylosing spondylitis (*N* = 11)	Psoriatic arthritis (*N* = 11)	Vasculitis* (*N* = 11)
**Demographics**							
Age (years)	59.2 ± 14.0	63.4 ± 12.3	53.2 ± 18.1	54.9 ± 14.3	52.9 ± 12.1	62.4 ± 8.1	53.4 ± 16.2
Male (%)	44 (37.9)	22 (38.6)	2 (18.2)	2 (18.2)	7 (63.6)	9 (81.8)	2 (18.2)
Ethnicity (%)							
White	101 (89.4)	52 (94.5)	9 (64.3)	10 (90.9)	9 (81.8)	11 (100.0)	10 (90.9)
Asian	4 (3.5)	2 (3.6)	2 (14.3)	0 (0.0)	0 (0.0)	0 (0.0)	0 (0.0)
Black	4 (3.5)	0 (0.0)	3 (21.4)	1 (9.1)	0 (0.0)	0 (0.0)	0 (0.0)
Not declared	4 (3.5)	1 (1.8)	0 (0.0)	0 (0.0)	2 (18.2)	0 (0.0)	1 (9.1)
Body mass index (kg/m^2^)	28.5 ± 6.9	29.2 ± 7.2	25.8 ± 5.0	24.2 ± 6.8	33.0 ± 8.0	30.2 ± 5.0	26.5 ± 4.6
**Comorbidity**							
Heart failure (%)	23 (20.0)	10 (17.9)	3 (20.0)	0 (0.0)	4 (36.4)	4 (36.4)	2 (18.2)
Ischaemic heart disease (%)	40 (34.5)	24 (42.1)	6 (40.0)	2 (18.2)	2 (18.2)	3 (27.3)	3 (27.3)
Stroke or TIA (%)	14 (12.1)	5 (8.8)	4 (26.7)	1 (9.1)	2 (18.2)	1 (9.1)	1 (9.1)
Peripheral vascular disease (%)	10 (8.6)	5 (8.8)	1 (6.7)	2 (18.2)	0 (0.0)	1 (9.1)	1 (9.1)
Diabetes (%)	11 (9.5)	7 (12.3)	0 (0.0)	1 (9.1)	3 (27.3)	0 (0.0)	0 (0.0)
Hypertension (%)	59 (50.9)	30 (52.6)	6 (40.0)	5 (45.5)	7 (63.6)	5 (45.4)	6 (54.5)
Hypercholesterolaemia (%)	55 (47.4)	29 (50.9)	5 (33.3)	6 (54.5)	5 (45.5)	4 (36.4)	6 (54.5)
Atrial fibrillation (%)	21 (18.1)	9 (15.8)	2 (13.3)	3 (27.3)	1 (9.1)	3 (27.3)	3 (27.3)
Chronic Obstructive Pulmonary Disease (%)	19 (16.4)	12 (21.1)	2 (13.3)	1 (9.1)	0 (0.0)	3 (27.3)	1 (9.1)
Current smoker (%)	11 (9.5)	7 (12.3)	1 (6.7)	1 (9.1)	2 (18.2)	0 (0.0)	0 (0.0)
Ex-smoker (%)	57 (49.1)	29 (50.9)	6 (40.0)	6 (54.5)	5 (45.5)	7 (63.6)	4 (36.4)
Family history of cardiovascular disease (%)	61 (52.6)	31 (54.4)	7 (46.7)	3 (27.3)	8 (72.7)	4 (36.4)	8 (72.7)
**Laboratory measurements**							
Haematocrit (L/L)	0.402 ± 0.043	0.403 ± 0.040	0.393 ± 0.038	0.372 ± 0.068	0.423 ± 0.040	0.420 ± 0.038	0.396 ± 0.042
Estimated glomerular filtration rate (mL/min/1.73m^2^)	77.4 ± 15.0	79.9 ± 12.4	74.7 ± 16.1	74.1 ± 19.6	77.2 ± 17.2	74.9 ± 17.5	74.0 ± 17
*ln*NT-proBNP	5.3 [4.6–7.0]	5.3 [4.9–7.6]	5.5 [4.3–6.7]	5.4 [5.1 − 5.6]	4.3 [4.0-5.5]	5.7 [4.9–6.4]	4.8 [ 4.4–5.7]
Raised NT-proBNP ≥125 pg/ml (%)	35 (67.3)	19 (76.0)	4 (57.1)	3 (75.0)	1 (25.0)	5 (83.3)	3 (50.0)
*ln*Hs-TnT	2.5 [1.8–3.0]	2.5 [1.9–2.9]	2.2 [2.0–2.4]	3.7 [2.5–5.4]	1.6 [1.1–2.4]	2.7 [2.5–3.3]	1.9 [1.7–2.5]
Raised Hs-TnT ≥ 14 ng/L (%)	20 (38.5)	11 (44.0)	1 (14.3)	3 (75.0)	1 (25.0)	3 (50.0)	1 (16.7)
*ln*GDF-15	7.3 [6.6–7.8]	7.3 [6.8–7.8]	6.5 [6.2–7.9]	7.3 [6.8 − 7.6]	7.2 [7.0–7.6]	7.3 [7.1–7.9]	7.2 [7.0–7.4]
**CMR characteristics**							
Left ventricle end diastolic volume index (ml/m^2^)	88.1 ± 27.9	88.2 ± 29.5	95.1 ± 33.3	80.4 ± 15.3	74.2 ± 17.7	87.7 ± 17.1	97.5 ± 36.7
Left ventricle end systolic volume index (ml/m^2^)	40.5 ± 26.4	43.6 ± 28.6	39.4 ± 24.1	29.5 ± 12	29.8 ± 12.4	36.9 ± 15.9	52.1 ± 41.9
Left ventricle ejection fraction (%)	57.0 ± 14.2	54.2 ± 15.3	60.9 ± 10.7	64.2 ± 9.7	60.2 ± 8.9	59.0 ± 12.4	52.9 ± 19.8
Left ventricle mass index (g/m^2^)	62.0 ± 19.7	62.7 ± 20.2	56.8 ± 17.6	61.6 ± 21.1	60.1 ± 14.9	73.9 ± 20.7	53.7 ± 17.6
Global longitudinal strain (%)	–15.2 ± 4.1	-14.1 ± 4.1	-16.1 ± 2.8	-17.4 ± 3.5	-15.9 ± 3.8	-15.4 ± 3.4	-16.7 ± 6.4
Right ventricle end diastolic volume index (ml/m^2^)	80.8 ± 17.8	79.0 ± 18	88.5 ± 20.6	82.9 ± 18.5	79.9 ± 18	81.2 ± 17.9	75.3 ± 9.9
Right ventricle end systolic volume index (ml/m^2^)	35.9 ± 12.8	36.9 ± 13.7	37.5 ± 12.9	35.8 ± 16.7	35.2 ± 10.4	33.2 ± 9.9	31.7 ± 12.9
Right ventricle ejection fraction (%)	56.5 ± 8.5	54.0 ± 8.7	59.4 ± 6.9	58.6 ± 11.2	56.3 ± 7	59.8 ± 5.3	58.1 ± 7.9
Left atrial area index (cm^2^/m^2^)	15.0 ± 3.7	15.2 ± 4.2	15.8 ± 2.7	15.1 ± 2.8	13.6 ± 2.6	13.8 ± 3.9	14.9 ± 3.5
Right atrial area index (cm^2^/m^2^)	12.7 ± 2.8	12.5 ± 3.2	12.2 ± 1.6	13.9 ± 2.9	13.2 ± 2.5	11.8 ± 2.1	13.7 ± 2.6
Infarct LGE (%)	18 (18.2)	11 (23.4)	1 (6.7)	1 (9.1)	2 (28.6)	2 (18.2)	1 (12.5)
Non-ischaemic LGE (%)	23 (23.2)	9 (19.1)	3 (20.0)	1 (9.1)	2 (28.6)	5 (45.5)	3 (37.5)
Myocardial ECV percentage (%)	27.8 ± 3.7	27.7 ± 3.9	28.3 ± 3	31.8 ± 3.8	26.1 ± 2.4	25.7 ± 3.4	27.8 ± 3.1

Values are n (%), mean ± SD, or median (IQR)

^Δ^ Of the l1 patients with systemic sclerosis, 4 had the limited form of the disease

* Of the 11 patients with vasculitis, 6 had ANCA-associated vasculitis, 2 had Behcet’s disease, 1 had Takayasu arteritis, 1 had Polyarteritis Nodosa and 1 had leukocytoclastic vasculitis

Missing values are shown in Supplementary Table 1

LGE = late gadolinium enhancement, NT-proBNP = N-terminal pro-B-type natriuretic peptide, hs-TnT = high sensitivity troponin T, GDF-15 = growth differentiation factor 15, ECV = extracellular volume

### Relationship between myocardial ECV and IMIDs

**Fig. 1:**
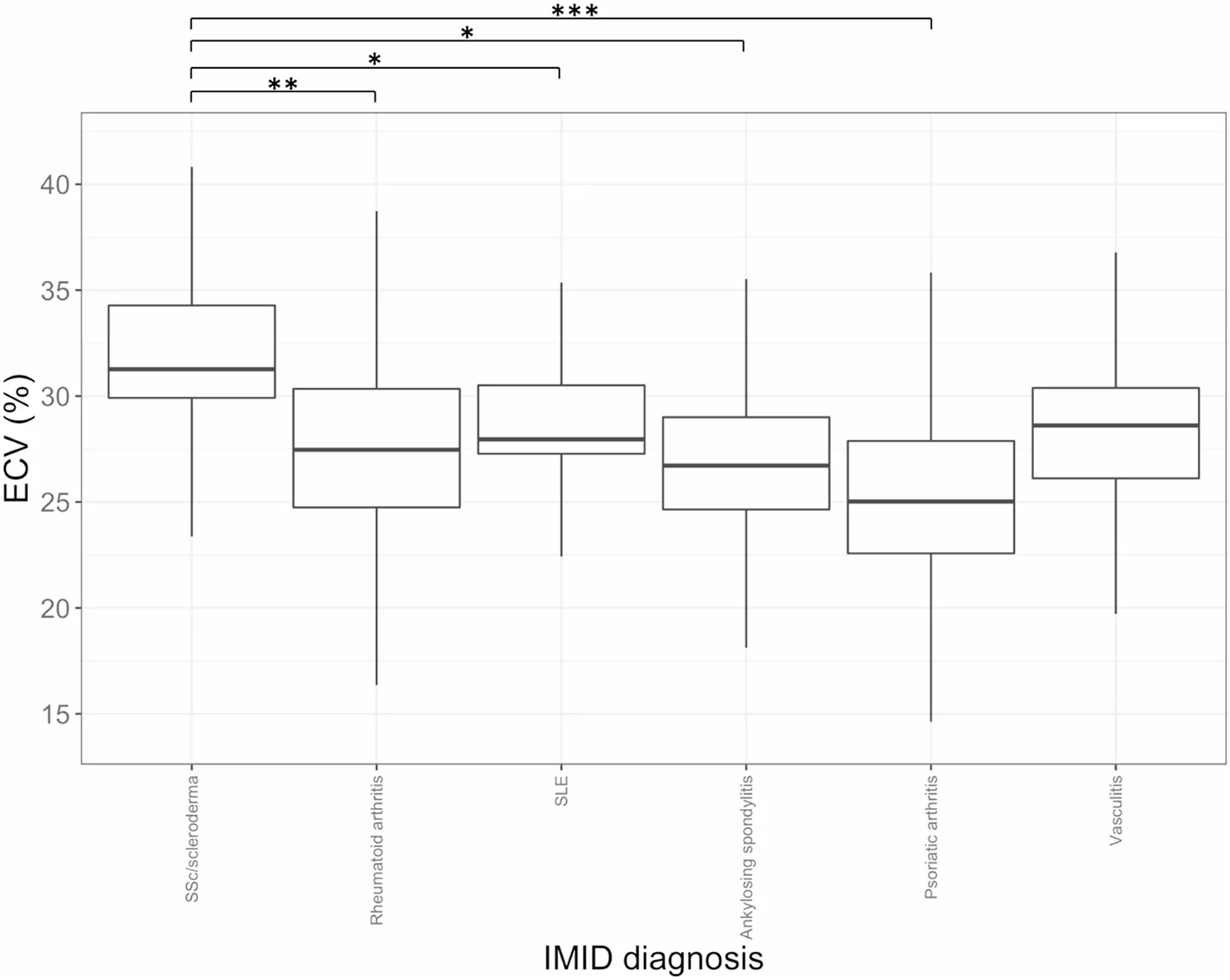
Boxplot of ECV by IMID diagnosis. Boxplot of ECV by IMID diagnosis. Median, first and third quartiles were calculated and pooled from all imputed datasets ANOVA and post hoc Tukey tests were calculated and pooled from all imputed datasets. Pooled ANOVA, F statistic 2.80, *P* = 0.015. Post hoc Tukey test * *p* < 0.05, ** *p* < 0.01, *** *p* < 0.001. ECV = extracellular volume, SLE = systemic lupus erythematosus, SSc = systemic sclerosis

**Fig. 2:**
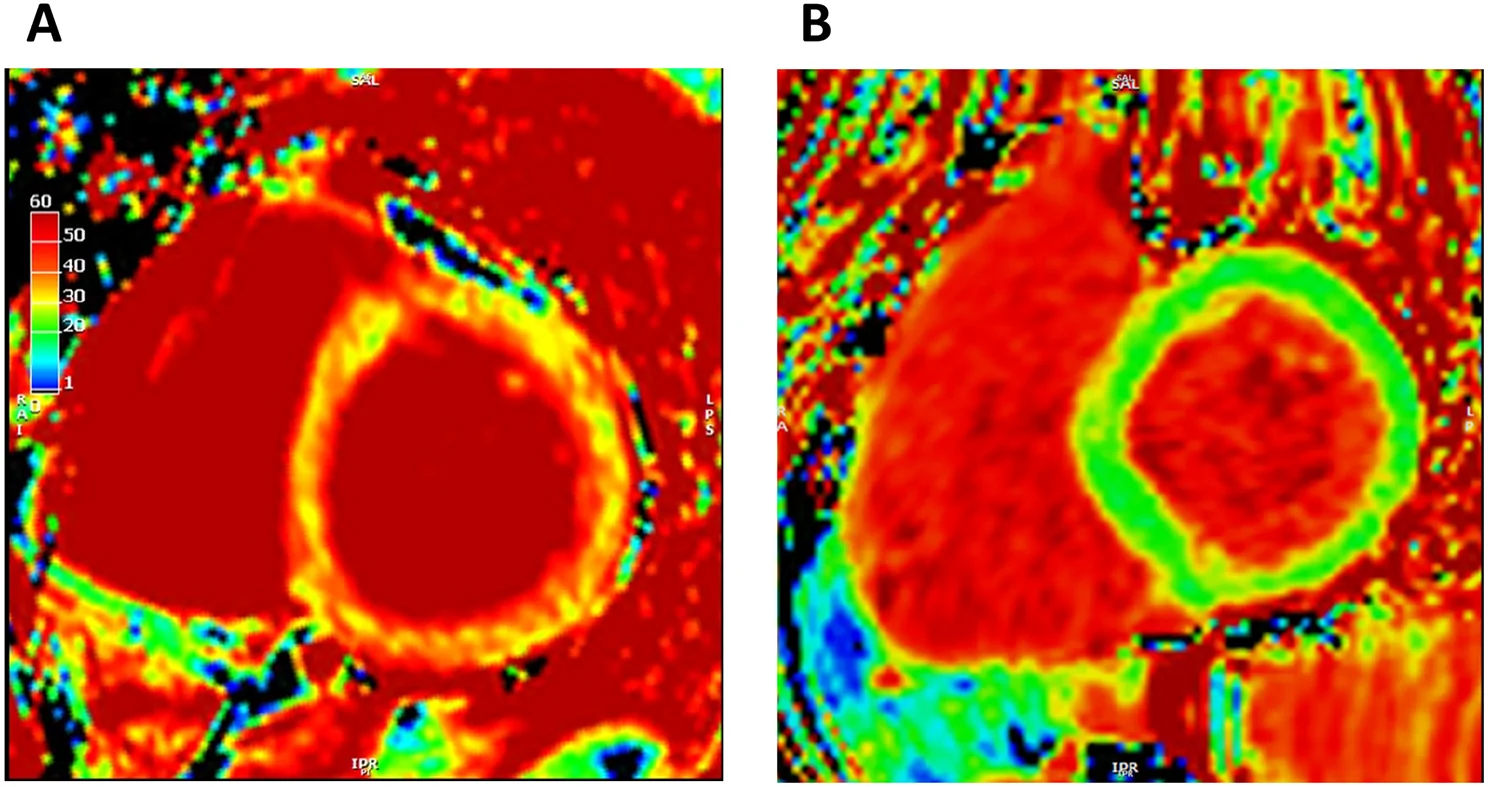
Illustrative ECV maps. **(A)** Patient with systemic sclerosis and ECV 35%. **(B)** Patient with rheumatoid arthritis and ECV 23%

Myocardial ECV varied significantly according to IMID diagnosis (pooled ANOVA F statistic 2.80, *P* = 0.015; Table 1; Fig. 1). ECV was significantly higher in SSc (mean ECV 31.8 ± standard deviation (SD) 3.8%) compared to other IMIDs: rheumatoid arthritis (mean ECV 27.7 ± 3.9%, *p* = 0.003), SLE (mean ECV 28.3 ± 3.0%, *p* = 0.031), ankylosing spondylitis (mean ECV 26.1 ± 2.4%, *p* = 0.014), and psoriatic arthritis (mean ECV 25.7 ± 3.4%, *p* < 0.001). There was a trend towards higher ECV in SSc compared to vasculitis (mean ECV 27.8 ± 3.1, *p* = 0.055). Illustrative ECV maps are shown in Fig. 2. An ECV cut-off of 30.5% was able to distinguish between SSc and other IMIDs with a sensitivity of 82% and specificity of 78% (Supplementary Fig. 5). There were no significant differences in other CMR measurements between IMID diagnoses.

### Determinants of myocardial ECV

**Fig. 3:**
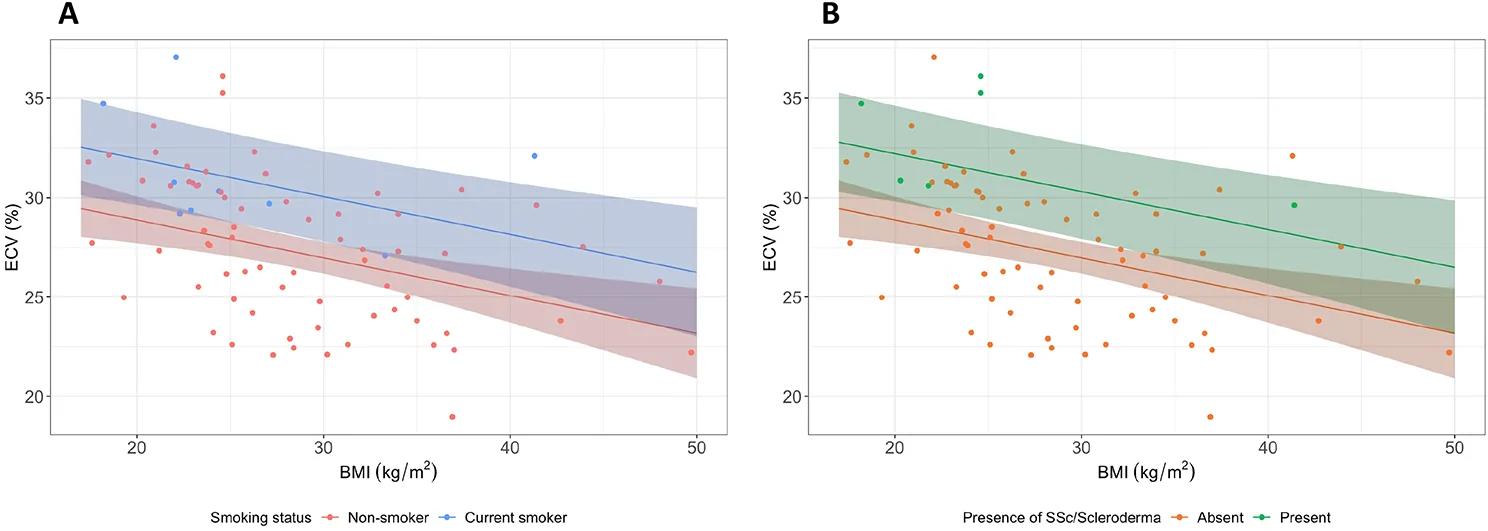
Multivariable linear model of determinants of ECV. **(A)** Scatterplot and regression line of correlation between ECV, BMI and smoking status (adjusted for no systemic sclerosis). Regression coefficients and 95% confidence intervals pooled from all imputed datasets, superimposed on data points from original unimputed dataset for reference. **(B)** Scatterplot and regression line of correlation between ECV, BMI and presence of systemic sclerosis (adjusted for non-smoker). Regression coefficients and 95% confidence intervals pooled from all imputed datasets, superimposed on data points from original unimputed dataset for reference

Univariable associations between baseline variables and myocardial ECV are presented in Supplementary Table 3. Independent determinants of ECV were presence of SSc, current smoking and BMI (Table 2; Fig. 3). Optimism adjusted pooled R^2^ and 95% confidence intervals for the multivariable model were 0.265 (0.142–0.391).

**Table 2:** Multivariable determinants of ECV

Term	Regression coefficient (standard error)	95% CI	t statistic	*p* value
(Intercept)	32.69 (1.51)	29.68–35.69	21.23	< 0.001
BMI	-0.19 (0.05)	-0.29 – -0.09	-3.75	< 0.001
Systemic Sclerosis	3.33 (1.26)	0.82–5.84	2.64	0.01
Current smoker	3.08 (1.18)	0.73–5.43	2.61	0.01

Adjusted pooled R^2^ and 95% confidence interval (CI) for model 0.269 (0.119–0.433)

Optimism adjusted pooled R^2^ and 95% CI for model 0.265 (0.142–0.391)

## Discussion

The principal findings of this study are that SSc is associated with a higher burden of myocardial fibrosis, as measured using ECV, compared to other IMIDs. Moreover, in patients with IMIDs, independent determinants of myocardial fibrosis were presence of SSc, smoking and BMI. Nevertheless, less than a quarter of the variability in ECV could be explained, indicating that determinants of myocardial fibrosis in patients with IMIDs remain largely unknown.

Cardiac involvement in IMIDs is related to either direct autoimmune mechanisms, manifesting as myocarditis and pericarditis, or accelerated coronary artery disease [3, 4]. LGE imaging with CMR has proven to be a powerful imaging modality in IMIDs. As well as being the reference standard for identifying myocardial infarction, which is typically seen in 10% of patients with IMIDs (18.2% in the current study) [23], LGE imaging is able to identify focal non-ischaemic myocardial fibrosis. Assumed to represent a sequela of direct inflammatory myocardial injury, non-ischaemic LGE is observed in advance of conventional structural or functional abnormalities, such as left ventricular dilatation or reduced LVEF.

However, the LGE technique is “not designed for quantifying fibrosis in non-infarcted myocardium and is not validated as a quantitative metric for this purpose” [17]. LGE detection of non-ischaemic fibrosis requires spatial heterogeneity. Gadolinium-based contrast agents accumulate in myocardium with expanded extracellular space. Visualisation of the accumulation requires myocardium with comparatively less expanded extracellular space for comparison. However, non-ischaemic myocardial fibrosis, i.e., excess collagen concentration in the myocardial interstitium, occurs in a continuum from mild to severe. In contrast, the CMR ECV technique simply quantifies the interstitial presence of gadolinium-based contrast agent relative to the plasma. Histological validation data show CMR ECV accurately and robustly measures non-ischaemic myocardial fibrosis [24, 25]. In keeping with these methodological factors, and demonstrating the diffuse nature of the associated myocardial fibrosis, SSc was associated with the highest burden of myocardial fibrosis as measured using ECV, but only 1 (9.1%) patient demonstrated non-ischaemic LGE.

Previous studies have shown ECV to be elevated in SSc compared to healthy controls [7, 15]. Findings have been variable in other IMID groups. In rheumatoid arthritis, ECV has been reported to be elevated [26], reduced [27] or similar [28] to healthy controls. Studies in SLE [29], ankylosing spondylitis [5], mixed inflammatory arthritis [30], and vasculitis [31] have been limited by small sample size. Our study is the first to measure ECV across the whole IMID spectrum. ECV was significantly elevated in SSc compared to other IMID diagnoses, suggesting SSc may involve pathophysiological mechanisms distinct from other IMIDs. Of the 11 patients with SSc, 4 had the limited form of the disease. In our cohort, ECV was similar in diffuse (31.4%) and limited (32.8%) forms of the disease. Prior studies have shown higher ECV in diffuse compared to limited forms of the disease, although ECV is elevated in both groups [32, 33]. Other studies have shown similar CMR measurements and non-ischaemic LGE in both groups [34]. As a result, subclinical diffuse myocardial fibrosis may be more common in the limited form of SSc than previously recognised.

Myocardial fibrosis, measured using ECV, is strongly predictive of adverse outcomes, including heart failure, arrythmia and death across a wide range of conditions [12–14]. There is emergent data to suggest this is true in IMIDs as well [35, 36]. Identifying determinants of ECV in patients with IMIDs is therefore important, and our study is the first to do so. SSc, current smoking, and BMI were independently predictive of ECV. SSc and smoking had a similarly large impact, associated with an average increase in ECV of ~ 3%. Interestingly there was a strong negative association between BMI and ECV, with a reduction in ECV of ~ 0.2% per unit increase in BMI. This finding has been replicated in large studies of pre-diabetic and diabetic patients [37] and in healthy patients without cardiovascular disease [38]. We cannot exclude the possibility that the relationship between BMI and ECV may be non-linear (U-shaped), with increased myocardial fibrosis noted at either end of the BMI spectrum. For instance, pre-clinical animal models demonstrate a strong association between obesity and histological fibrosis [39–41]. Further investigation is required to understand the mechanisms. Although circulating cardiac biomarkers are not specific to myocardial fibrosis, previous studies have noted a positive association between ECV and NT-proBNP [42], hsTnT [43] and GDF-15 [44]. We noted significant univariable associations between these cardiac biomarkers and ECV, but no association after multivariable adjustment. We hypothesise that this may be due to the dominant effects of SSc and smoking masking the effects of less significant variables, or alternatively that the study was underpowered to detect smaller effect sizes.

The magnitude of the impact that these variables have on ECV are prognostically relevant; for example in a cohort of patients with heart failure and preserved ejection fraction, a 1% increase in ECV was associated with a 7% increase in the hazard of heart failure hospitalisation or death after multivariable adjustment [13]. Although we are cautious to avoid conflating association with causation, it is noteworthy that smoking and BMI are both modifiable risk factors and hence lifestyle interventions aimed at smoking cessation and reasonable weight gain may reduce myocardial fibrosis and adverse cardiovascular outcomes in this cohort. This hypothesis warrants further investigation. Alternatively, low BMI may be a marker of advanced disease and ECV may simply be correlated with worse disease rather than BMI per se. Despite extensive phenotyping, our multivariable model was only able to explain approximately a quarter of the variability in ECV (optimism adjusted R^2^ 0.265). This reinforces the fact that mechanisms responsible for diffuse myocardial fibrosis remain largely unexplained. At least some of this variation may be explained by measures of disease activity and detailed medication history, which were not available in our cohort. Previous studies in SSc have shown that ECV is positively correlated with disease activity, measured by Valentini disease activity index [32], modified Rodnan skin score [15, 32], and presence of digital ulceration [15]. In addition, ECV is associated with worse cardiovascular outcomes in SSc [35]. We therefore hypothesise that improved disease management may associate with reduced myocardial fibrosis and improved cardiovascular outcomes in IMIDs, but this requires further investigation in dedicated trials.

Myocardial fibrosis is a promising therapeutic target in patients with IMIDs, particularly SSc. Pirfenidone, an anti-fibrotic agent without haemodynamic effect, was associated with a significant reduction in myocardial fibrosis, measured using ECV, and a significant reduction in NT-proBNP, in patients with heart failure with preserved ejection fraction [45]. Although pirfenidone has been investigated in SSc-associated interstitial lung disease [46], its effect on myocardial fibrosis in SSc and other IMIDs requires further investigation.

Our study has several limitations. The sample size is small with only small numbers of patients with certain IMIDs, hence the study is not powered to identify determinants of myocardial fibrosis with smaller effect sizes. In addition, the percentage of missing data in the study was relatively high although this was addressed with multiple imputation in accordance with best practice [20]. Due to logistics, it was not possible to measure cardiac biomarkers in all patients. ECV made up most of the remaining missing data. Reasons may include missing pre- or post-contrast T1 maps (technical difficulty, missing images due to claustrophobia, non-contrast scan indications, contrast contraindications) or haemolysed same-day haematocrit. Participants underwent CMR for clinical indications and may not be representative of IMID populations in the community. Information on IMID disease severity, measures of disease activity and medication history were not available. We cannot therefore exclude the possibility that the observed differences in ECV between IMIDs were due to disease stage. Finally elevated myocardial ECV may be due to increased collagen deposition (myocardial fibrosis) or oedema (active inflammation), and T2 imaging data was not available to quantify the oedema component. Nonetheless, we hypothesise that fibrosis is likely to be the dominant mechanism in this cohort of stable outpatients.

## Conclusion

In conclusion, SSc is associated with a higher burden of myocardial fibrosis compared to other IMIDs. In patients with IMIDs, independent determinants of myocardial fibrosis included SSc, smoking and BMI.

## Electronic supplementary material

Below is the link to the electronic supplementary material.

Supplementary Material 1

## Data Availability

No datasets were generated or analysed during the current study.

## Abbreviations

BMI: Body mass index
COPD: Chronic obstructive pulmonary disease
CMR: Cardiovascular magnetic resonance
ECV: Extracellular volume
GDF-15: Growth differentiation factor 15
GLS: Global longitudinal strain
hsTnT: High sensitivity troponin T
IMID: Immune–mediated inflammatory disease
LGE: Late gadolinium enhancement
*ln*: Natural logarithm
LVEF: Left ventricle ejection fraction
NT-proBNP–: N–terminal pro–B–type natriuretic peptide
SSc: Systemic sclerosis
SLE: Systemic lupus erythematosus
TIA: Transient ischaemic attack
