# Supplementary material for: Determinants of myocardial fibrosis in patients with immune-mediated inflammatory diseases
Source: Adv Rheumatol. 2025 Jun 14;65:28. doi: 10.1186/s42358-025-00451-w (PMC12167332; doi:10.1186/s42358-025-00451-w)
Supplement: Supplementary file 1 [file 42358_2025_451_MOESM1_ESM.docx]

**Determinants of myocardial fibrosis in patients with immune-mediated inflammatory diseases**

# Supplemental Material

**Supplementary Table 1. Missing values**

|  | **Overall**  **(N=116)** |  | **Overall**  **(N=116)** |
| --- | --- | --- | --- |
| **Demographics** |  | **Laboratory measurements** |  |
| Age (%) | 0 (0.0) | Estimated glomerular filtration rate (%) | 9 (7.8) |
| Male (%) | 0 (0.0) | NT-proBNP (%) | 64 (55.2) |
| Ethnicity (%) | 3 (2.6) | Hs-TnT (%) | 64 (55.2) |
| Body mass index (%) | 6 (5.2) | GDF-15 (%) | 66 (56.9) |
| IMID diagnosis (%) | 0 (0.0) |  |  |
|  |  | **CMR characteristics** |  |
| **Comorbidity** |  | Left ventricle end diastolic volume index (%) | 15 (12.9) |
| Heart failure (%) | 1 (0.9) | Left ventricle end systolic volume index (%) | 15 (12.9) |
| Ischaemic heart disease (%) | 0 (0.0) | Left ventricle ejection fraction (%) | 15 (12.9) |
| Stroke or TIA (%) | 0 (0.0) | Left ventricle mass index (%) | 16 (13.8) |
| Peripheral vascular disease (%) | 0 (0.0) | Global longitudinal strain (%) | 17 (14.7) |
| Diabetes (%) | 0 (0.0) | Right ventricle end diastolic volume index (%) | 17 (14.7) |
| Hypertension (%) | 0 (0.0) | Right ventricle end systolic volume index (%) | 17 (14.7) |
| Hypercholesterolaemia (%) | 0 (0.0) | Right ventricle ejection fraction (%) | 17 (14.7) |
| Atrial fibrillation (%) | 0 (0.0) | Left atrial area index (%) | 11 (9.5) |
| Chronic Obstructive Pulmonary Disease (%) | 0 (0.0) | Right atrial area index (%) | 11 (9.5) |
| Current smoker (%) | 0 (0.0) | Infarct LGE (%) | 17 (14.7) |
| Ex-smoker (%) | 0 (0.0) | Non-ischaemic LGE (%) | 17 (14.7) |
| Family history of cardiovascular disease (%) | 0 (0.0) | Myocardial ECV percentage (%) | 33 (28.4) |

Missing values are n (%).

Overall percentage of missing data 11.4%.

**Supplementary figure 1.** Satisfactory imputation of ECV using MICE package in R.

**Supplementary figure 2.** Density plot for imputed ECV values using MICE package in R.

**Supplementary figure 3.** Strip plot for imputed ECV values using MICE package in R.

**Supplementary figure 4.** Plot to assess multivariable linear model assumptions. Plot shown for second imputed dataset for demonstration purposes**.**

**Supplementary Table 2. CMR scan indications**

| **Scan indications** | **Number of patients (%)** |
| --- | --- |
| Ischaemic heart disease | 34 (29.3 %) |
| Heart failure | 27 (23.3 %) |
| Cardiomyopathy | 16 (13.8 %) |
| Myocarditis | 14 (12.1 %) |
| Aortic disease | 9 (7.8 %) |
| Other | 8 (6.9 %) |
| Arrhythmia | 5 (4.3 %) |
| Valvular heart disease | 3 (2.6 %) |

**Supplementary figure 5.** Receiver operating characteristic (ROC) curve of sensitivity and specificity of ECV for classifying SSc vs other IMID diagnoses.

**Supplementary Table 3. Univariable associations between candidate variables and ECV**

| **Variable** | **Intercept** | **Regression coefficient (standard error)** | **95% CI** | **t statistic** | **P value** | **Adjusted R^2^** |
| --- | --- | --- | --- | --- | --- | --- |
| Age | 29.30 | -0.02 (0.03) | -0.08 – 0.04 | -0.77 | 0.443 | 0.002 |
| Female sex | 27.03 | 1.41 (0.81) | -0.20 – 3.02 | 1.74 | 0.086 | 0.026 |
| White ethnicity | 29.49 | -1.80 (1.21) | -4.22 – 0.61 | -1.48 | 0.141 | 0.018 |
| BMI | 34.24 | -0.22 (0.06) | -0.33 – -0.11 | -4.04 | <0.001* | 0.157 |
| SSc | 27.51 | 4.19 (1.36) | 1.47 – 6.91 | 3.07 | 0.003* | 0.099 |
| Ischaemic heart disease | 27.79 | 0.32 (0.84) | -1.35 – 1.99 | 0.39 | 0.701 | -0.005 |
| Stroke or TIA | 27.77 | 1.15 (1.29) | -1.42 – 3.71 | 0.89 | 0.376 | 0.004 |
| Peripheral vascular disease | 27.92 | -0.21 (1.56) | -3.32 – 2.90 | -0.14 | 0.893 | -0.004 |
| Diabetes | 27.94 | -0.39 (1.35) | -3.07 – 2.28 | -0.29 | 0.771 | -0.006 |
| Hypertension | 28.21 | -0.60 (0.79) | -2.17 – 0.98 | -0.75 | 0.454 | -0.001 |
| Hypercholesterolaemia | 28.23 | -0.68 (0.80) | -2.27 – 0.91 | -0.85 | 0.397 | 0.002 |
| Atrial Fibrillation | 28.10 | -1.07 (0.96) | -2.98 – 0.83 | -1.12 | 0.267 | 0.004 |
| COPD | 27.71 | 1.20 (1.09) | -0.97 – 3.37 | 1.10 | 0.275 | 0.008 |
| Current smoker | 27.62 | 3.05 (1.29) | 0.48 – 5.62 | 2.36 | 0.020* | 0.049 |
| *ln* NTproBNP | 26.70 | 0.21 (0.26) | -0.30 – 0.73 | 0.82 | 0.416 | 0.006 |
| *ln* hsTn | 26.44 | 0.59 (0.39) | -0.20 – 1.38 | 1.50 | 0.139 | 0.034 |
| *ln* GDF15 | 26.79 | 0.15 (0.61) | -1.06 – 1.37 | 0.26 | 0.800 | 0.000 |
| LVEF | 29.50 | -0.03 (0.03) | -0.09 – 0.03 | -0.92 | 0.363 | 0.007 |
| GLS | 28.99 | 0.07 (0.1) | -0.12 – 0.26 | 0.74 | 0.463 | -0.001 |
| Indexed Myocardial Mass | 27.70 | 0.00 (0.02) | -0.04 – 0.04 | 0.16 | 0.875 | -0.006 |
| Infarct LGE mass percentage | 27.84 | 0.03 (0.06) | -0.09 – 0.14 | 0.48 | 0.634 | -0.005 |
| Non-ischaemic LGE mass percentage | 27.99 | -0.12 (0.19) | -0.50 – 0.25 | -0.65 | 0.515 | -0.004 |

ECV = extracellular volume. BMI = body mass index. SSc = systemic sclerosis. TIA = transient ischaemic attack. COPD = chronic obstructive pulmonary disease. *ln* = natural logarithm. NT-proBNP = N-terminal pro-B-type natriuretic peptide. hsTn = high sensitivity troponin. GDF-15 = growth differentiation factor-15. LVEF = left ventricle ejection fraction. GLS = global longitudinal strain. LGE=late gadolinium enhancement. • p<0.05
